# The application of low-dose rivaroxaban in elderly patients with atrial fibrillation

**DOI:** 10.3389/fphar.2026.1756880

**Published:** 2026-05-12

**Authors:** Zhen Wang, Xinyi Dai, Zhengmei Huang, Jingtang Hu, Jiangyan Qu, Peng Wang, Qian Chen, Xiong Wang

**Affiliations:** Department of Cardiology, The General Hospital of Western Theater Command, Chengdu, China

**Keywords:** anticoagulation, non-valvular atrial fibrillation, elderly patients, low-dose rivaroxaban, stroke prevention

## Abstract

**Background:**

The optimal anticoagulation strategy for stroke prevention in very elderly patients (≥80 years) with non-valvular atrial fibrillation (NVAF) remains challenging due to the high risks of both thromboembolism and bleeding. This study aimed to evaluate the effectiveness and safety of low-dose rivaroxaban (10 mg) in this specific population.

**Methods:**

This single-center, real-world cohort study enrolled 1,005 patients aged ≥80 years with NVAF between January 2021 and December 2023. Patients were categorized into three groups based on their antithrombotic regimen: a non-anticoagulation group (n = 177), a low-dose rivaroxaban group (10 mg, n = 168), and a conventional anticoagulation group (n = 660). The primary efficacy outcome was stroke, and the primary safety outcome was major bleeding. Outcomes were analyzed using multivariable Cox regression models and competing risk models to account for death as a competing event where appropriate.

**Results:**

Compared with no anticoagulation, low-dose rivaroxaban was associated with significantly lower risks of stroke (hazard ratio (HR) 0.20, 95% confidence interval (CI) 0.05–0.78; P = 0.0201), all-cause mortality (HR 0.28, 95% CI 0.13–0.60; P = 0.0012), and cardiac death (HR 0.31, 95% CI 0.17–0.81; P = 0.0170), with efficacy comparable to conventional anticoagulation. Direct comparison showed no significant differences in efficacy outcomes between the low-dose and conventional anticoagulation groups. Moreover, the risk of major bleeding was numerically lower with low-dose rivaroxaban than with conventional anticoagulation (HR 2.01 for conventional vs. low-dose, 95% CI 0.93–4.12; P = 0.0856).

**Conclusion:**

In very elderly patients (≥80 years) with NVAF, low-dose rivaroxaban (10 mg) was associated with similar effectiveness to conventional anticoagulation in preventing stroke and reducing mortality, while showing a potential safety advantage with a numerically lower risk of major bleeding. These findings support the use of low-dose rivaroxaban as a favorable individualized antithrombotic strategy for this vulnerable population.

## 1 Introduction

Atrial fibrillation (AF), the most common sustained cardiac arrhythmia worldwide, poses a significant and growing global health burden. Its prevalence increases markedly with age, with a reported lifetime risk approaching one-third in adults (Hindricks et al., 2020; Kornej et al., 2020). As the global population ages, the number of individuals affected by AF has risen substantially, reaching nearly 60 million in 2019 and resulting in considerable mortality and disability (Li et al., 2022). Projections suggest AF could be attributable to 2.5 million deaths between 2030 and 2034 (Li et al., 2022), highlighting the urgent need for effective management strategies.

Preventing thromboembolic complications, particularly stroke, is a central goal in AF management. Advanced age is a powerful risk factor for ischemic stroke and systemic embolism in AF patients (Camm et al., 2012; Friberg et al., 2012), and the proportion of strokes attributable to AF rises with age (Wolf et al., 1991). This is formally recognized in the CHA_2_DS_2_-VASc score, where age ≥75 years contributes significantly. However, the benefit of anticoagulation is counterbalanced by a parallel increase in bleeding risk. Older adults, especially those over 85, experience substantially higher rates of intracranial hemorrhage (ICH) during anticoagulant therapy, such as with warfarin (Fang et al., 2004). Thus, stroke prevention in the elderly AF population remains a delicate balancing act between thromboembolic protection and hemorrhagic risk.

The advent of direct oral anticoagulants (DOACs) marked a paradigm shift in stroke prevention, demonstrating superior or non-inferior efficacy and safety compared to vitamin K antagonists (VKAs) in large clinical trials (Connolly et al., 2009). Despite this overall success, important questions remain regarding their use in specific subgroups. Notably, patients aged ≥75 years were underrepresented in the pivotal DOAC trials. Given their elevated risks for both thrombosis and bleeding, the optimal anticoagulant strategy in this demographic is less defined. Real-world data further complicate the picture, indicating that inappropriate DOAC dosing—often underdosing due to bleeding concerns—occurs in a significant proportion (up to 18%) of elderly patients, with advanced age itself being a key predictor (Guenoun et al., 2023).

A critical analysis of DOAC performance in the elderly reveals nuances not apparent in the broader trial populations. While DOACs generally maintained a favorable safety profile compared to VKAs, the magnitude of benefit, particularly regarding ICH risk reduction, may not be uniform across all DOACs in older adults. Some evidence suggests that the ICH risk advantage of rivaroxaban over VKAs observed in the general population may be attenuated in the elderly (Okumura et al., 2020; Granger et al., 2011; Patel et al., 2011; Giugliano et al., 2013). This potential age-related alteration in the risk-benefit profile underscores the clinical dilemma and justifies the exploration of tailored dosing strategies.

Bleeding concerns profoundly influence therapeutic decisions. A European survey found that over 10% of physicians consider advanced age a major deterrent to anticoagulation (Fumagalli et al., 2017). In this context, dose reduction emerges as an intuitive, though not fully evidence-based, approach to mitigating bleeding risk in vulnerable elderly patients. This study therefore aims to evaluate the effectiveness and safety of low-dose rivaroxaban (10 mg) specifically in very elderly patients (≥80 years) with NVAF, a population at the extreme end of this risk-benefit continuum.

## 2 Methods

### 2.1 Trial design

This was a single-center cohort study conducted at the General Hospital of Western Theater Command. The ethics committee at the General Hospital of Western Theater Command approved the protocol. All patients provided written informed consent. Patient enrollment began in January 2021 and concluded in December 2023, with a follow-up period of 12 months for all patients.

### 2.2 Participants

**FIGURE 1:**
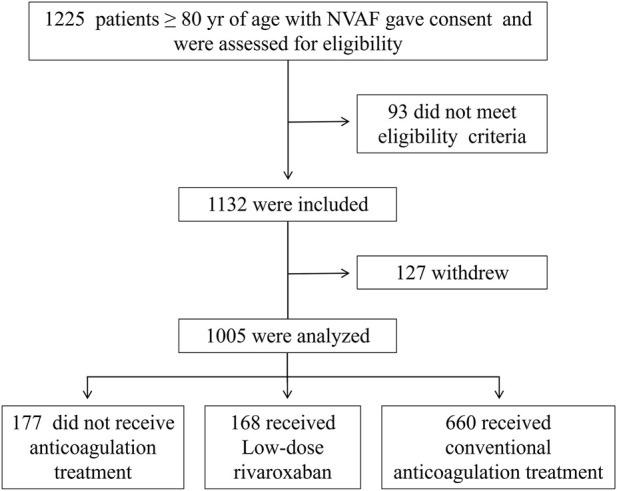
Study flowchart. NVAF, Non-Valvular Atrial Fibrillation.

This was an observational study conducted in a real-world setting to compare different anticoagulation regimens in elderly Chinese patients (≥80 years of age) with non-valvular atrial fibrillation (NVAF). A total of 1,248 patients were initially screened. After applying the eligibility criteria and conducting follow-up visits, 1,005 patients were included in the final analysis. The detailed patient selection process is shown in Figure 1.

Inclusion criteria were: (1) age ≥80 years; (2) a confirmed diagnosis of non-valvular atrial fibrillation (paroxysmal, persistent, or permanent); Exclusion criteria were: (1) valvular atrial fibrillation (moderate to severe mitral stenosis or mechanical heart valves); (2) need for anticoagulation for conditions other than AF; (3) contraindications to anticoagulation; (4) malignant tumor; (5) history of intracranial hemorrhage; (6) use of antiplatelet therapy; (7) life expectancy less than 12 months. Anticoagulation treatment plans for stroke prevention were prescribed by physicians based on comprehensive clinical assessments. The conventional anticoagulation group included patients receiving standard-dose anticoagulation therapy as prescribed for stroke prevention, comprising: standard-dose rivaroxaban (15 mg once daily), standard-dose edoxaban (30 mg or 60 mg once daily), and warfarin (dose-adjusted to target international normalized ratio 2.0–3.0). The primary efficacy endpoint was stroke, and the primary safety endpoint was major bleeding according to the definition of the International Society on Thrombosis and Haemostasis. The Helsinki Declaration was strictly adhered to in this study. All patients provided written informed consent, and their data were anonymized during collection, analysis, and reporting.

### 2.3 Outcomes

The primary efficacy outcome of this study was stroke, including both ischemic and hemorrhagic stroke. Stroke was defined as a rapid onset focal neurological deficit of presumed vascular origin, lasting for ≥24 h or resulting in death, with or without confirmation by neuroimaging. Secondary efficacy outcomes included: All-cause mortality: Death from any cause during the follow-up period; Cardiac death: Death resulting from cardiovascular causes, including fatal stroke, fatal myocardial infarction, sudden cardiac death, and death due to heart failure. The primary safety outcome was major bleeding, defined according to the criteria of the International Society on Thrombosis and Haemostasis (ISTH) as: fatal bleeding, symptomatic bleeding in a critical area or organ (such as intracranial, intraspinal, intraocular, retroperitoneal, intra-articular, pericardial, or intramuscular with compartment syndrome), bleeding causing a fall in hemoglobin level of 20 g L^-1^ or more, or leading to transfusion of two or more units of whole blood or red cells.

All outcomes were adjudicated by a clinical events committee based on medical records, discharge summaries, and, if necessary, death certificates. Patients were followed up for 12 months *via* outpatient clinic visits or telephone interviews to capture all outcome events.

### 2.4 Statistical analysis

Data are presented as median and interquartile range [IQR] for continuous variables with non-normal distribution, and as counts and percentages for categorical variables. Normality was assessed using the Shapiro-Wilk test. For continuous variables, the Kruskal–Wallis test was used to compare differences among the three groups. For categorical variables, the Chi-square test was used to compare differences in proportions among the three groups.

Cumulative incidence functions were estimated for outcomes in the presence of competing risks, specifically for major bleeding, stroke (with death as a competing event) and cardiac death (with non-cardiac death as a competing event). Differences in cumulative incidence functions between groups were compared. For all-cause mortality, where no competing risk exists, the Kaplan-Meier method with log-rank test was used. To evaluate the associations between anticoagulation strategies and clinical outcomes, we used multivariable Cox proportional hazards regression models. Modes were adjusted for a more comprehensive set of clinically relevant covariates selected based on a combination of clinical relevance, baseline imbalance, and established risk factors from the literature. The final list of covariates included varied slightly depending on the outcome being analyzed, but was generally drawn from the following: age, sex, estimated glomerular filtration rate, left atrial diameter, hemoglobin, coronary heart disease, diabetes, hypertension, use of nonsteroidal anti-inflammatory drugs, and use of angiotensin-converting enzyme inhibitors. The specific covariates adjusted for in each analysis are indicated in the footnotes of the respective tables (Tables 1, 2). For major bleeding and stroke, we accounted for the competing risk of death. For cardiac death, non-cardiac death was treated as a competing risk. Thus, hazard ratios were estimated using the competing risk analysis. For all Cox proportional hazards models and competing risk models, we tested the proportional hazards assumption. For standard Cox models, we used Schoenfeld residuals and for competing risk models, we included a time-covariate interaction term. The proportional hazards assumption was considered satisfied if the global Schoenfeld test was non-significant (P > 0.05) or if time-covariate interactions were non-significant (P > 0.05). All models presented in this study satisfied the proportional hazards assumption. All of the analyses were performed with the statistical software packages R (http://www.R-project.org, The R Foundation) and EmpowerStats (http://www.empowerstats.com, X&Y Solutions, Inc., Boston, MA). A two-sided significance level of 0.05 was used to evaluate statistical significance.

**TABLE 1:** Baseline characteristics.

Characteristic	Non anticoagulant	Low-dose rivaroxaban	Conventional anticoagulation	*P* Value
n	177	168	660	​
AGE, yrs, median (IQR)	86.00 (82.00–89.00)	86.50 (82.00–89.00)	83.00 (81.00–86.00)	<0.001
Male, n (%)	87 (49.15%)	72 (42.86%)	312 (47.27%)	0.472
BMI, kg/m2, median (IQR)	22.54 (18.81–26.12)	22.71 (20.00–25.40)	23.30 (20.44–26.01)	0.204
Persistent atrial fibrillation, n (%)	63 (35.59%)	108 (64.29%)	410 (62.12%)	<0.001
SBP, mmHg, median (IQR)	126.00 (105.00–146.00)	131.00 (121.00–138.25)	128.00 (115.00–142.00)	0.222
Smoker, n (%)	60 (33.90%)	39 (23.21%)	170 (25.76%)	0.862
Drinking, n (%)	42 (23.73%)	39 (23.21%)	166 (25.15%)	0.838
Heart failure, n (%)	87 (49.15%)	84 (50.00%)	302 (45.90%)	0.536
Coronary heart disease, n (%)	102 (57.63%)	81 (48.21%)	292 (44.24%)	0.006
Stroke, n (%)	51 (28.81%)	45 (26.79%)	190 (28.88%)	0.862
Diabetes, n (%)	33 (18.64%)	54 (32.14%)	138 (20.91%)	0.003
Hypertension, n (%)	99 (55.93%)	123 (73.21%)	420 (63.64%)	0.004
LVEF, %, median (IQR)	55.00 (49.00–60.00)	55.00 (50.00–60.00)	56.00 (47.00–60.00)	0.995
LA diameter, mm, median (IQR)	41.50 (38.75–44.25)	44.00 (40.00–51.00)	45.00 (39.00–49.00)	0.010
CHADS2 -VA score, median (IQR)	5.00 (4.00–6.00)	5.00 (4.00–6.00)	5.00 (4.00–6.00)	0.052
eGFR, %, median (IQR)	56.00 (48.26–75.52)	57.90 (40.96–69.20)	62.56 (48.05–73.60)	0.009
Hemoglobin, g/L, median (IQR)	121.00 (106.00–133.00)	123.00 (110.00–136.00)	127.00 (117.00–137.25)	<0.001
NSAID, n (%)	51 (28.81%)	3 (1.82%)	18 (2.73%)	<0.001
ACEI, n (%)	42 (23.73%)	21 (12.50%)	180 (27.36%)	<0.001
ARB, n (%)	54 (30.51%)	60 (35.71%)	228 (34.55%)	0.530
Statin, n (%)	123 (69.49%)	126 (75.00%)	478 (72.42%)	0.518

IQR, interquartile range; SBP, systolic blood pressure; BMI, body mass index; LVEF, left ventricular ejection fraction; ACEI, angiotensin-converting enzyme inhibitors; eGFR, estimated glomerular filtration rate; NSAID, nonsteroidal antiinflammatory drugs; ARB, angiotensin receptor blocker.

**TABLE 2:** Primary and secondary efficacy and safety end points.

Multivariate cox regression analysis							
Outcome	Non-anticoagulation	Low-dose rivaroxaban			Conventional anticoagulation		
​	Patients with event (%)	Patients with event (%)	Hazard ratio (95% Cl)	P value	Patients with event (%)	Hazard ratio (95% Cl)	P value
Major bleeding	6 (3.39%)	10 (5.95%)	1.80 (1.04, 5.08)	0.0263	56 (8.48%)	2.64 (1.12, 6.24)	0.0266
Stroke	12 (6.78%)	3 (1.79%)	0.25 (0.07, 0.90)	0.0343	10 (1.52%)	0.21 (0.09, 0.50)	0.0004
All cause death	30 (16.96%)	9 (5.36%)	0.28 (0.13, 0.60)	0.0012	40 (6.06%)	0.32 (0.19, 0.52)	<0.0001
Cardiac death	21 (11.86%)	6 (3.57%)	0.28 (0.11, 0.70)	0.0067	24 (3.64%)	0.28 (0.15, 0.52)	<0.0001
Competing risk analysis							
Major bleeding	6 (3.39%)	10 (5.95%)	1.97 (1.14, 5.43)	0.0291	56 (8.48%)	2.10 (1.18, 4.91)	0.0280
Stroke	12 (6.78%)	3 (1.79%)	0.20 (0.05, 0.78)	0.0201	10 (1.52%)	0.21 (0.09, 0.49)	0.0004
Cardiac death	21 (11.86%)	6 (3.57%)	0.31 (0.17, 0.81)	0.0170	24 (3.64%)	0.28 (0.15, 0.52)	<0.0001

All adjusted for age, gender, eGFR, LA diameter, hemoglobin, coronary heart disease, NSAID. eGFR, estimated glomerular filtration rate; ACEI, angiotensin-converting enzyme inhibitors.

## 3 Results

A total of 1,005 patients with non-valvular atrial fibrillation aged ≥80 years were included in this study. According to the anticoagulation regimen, the patients were categorized into three groups: a non-anticoagulation group (n = 177), a low-dose rivaroxaban group (10 mg, n = 168), and a conventional anticoagulation group (n = 660). The baseline characteristics of the patients are summarized in Table 1. Patients in the conventional anticoagulation group were relatively younger and exhibited higher levels of estimated glomerular filtration rate (eGFR) and hemoglobin. Those in the non-anticoagulation group were more frequently diagnosed with paroxysmal atrial fibrillation and coronary heart disease, and had a greater use of nonsteroidal anti-inflammatory drugs. They also showed a smaller left atrial anterior-posterior diameter. In contrast, patients receiving low-dose rivaroxaban had a higher prevalence of hypertension and diabetes, along with lower use of angiotensin-converting enzyme inhibitors (ACEIs).

**FIGURE 2:**
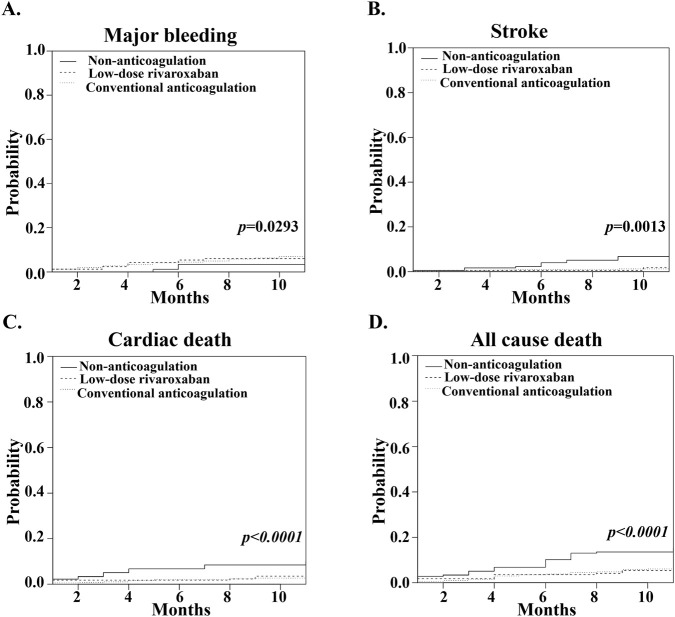
Cumulative incidence functions for efficacy and safety outcomes. Panels show cumulative incidence functions for **(A)** major bleeding **(B)** stroke **(C)** cardiac death, and **(D)** all-cause mortality. For major bleeding and stroke, death from any cause was treated as a competing event. For cardiac death, non-cardiac death was treated as a competing event. All-cause mortality is presented as a Kaplan-Meier curve.

For the safety endpoints, there were 6 events of major bleeding (3.39% per patient-year) in the non-anticoagulation group, 10 (5.95% per patient-year) in the low-dose rivaroxaban group (HR, 1.80; 95% CI, 1.04 to 5.08; P = 0.0263 for superiority), and 56 (8.48% per patient-year) in the conventional anticoagulation group (HR, 2.64; 95% CI, 1.12 to 6.24; P = 0.0266 for superiority). Given the extremely high risk of death among the elderly patients which acts as a competing event for major bleeding, we conducted a competitive analysis. Compared with no anticoagulation, low-dose rivaroxaban (10 mg) remained significantly associated with an increased risk of major bleeding (HR, 1.97; 95% CI, 1.14 to 5.43; P = 0.0291 for superiority). Conventional anticoagulation also remained significantly associated with an increased risk of major bleeding compared to no anticoagulation (HR, 2.10; 95% CI, 1.18 to 4.91; P = 0.0280 for superiority). The results of this competing risk analysis are largely consistent with our original findings, confirming the robustness of our conclusions (Table 2; Figure 2A).

Regarding the efficacy endpoint, a total of 25 events of stroke were reported. 10 patients (6.78% per patient-year) in the non-anticoagulation group had a stroke. In the low-dose rivaroxaban group, this number was 3 (1.79% per patient-year) (HR, 0.25; 95% CI, 0.07 to 0.90; P = 0.0343 for superiority) and in conventional anticoagulation group, it was 10 (1.52% per patient-year) (HR, 0.21; 95% CI, 0.09 to 0.50; P = 0.0004 for superiority) (Table 2). Death is also a competing factor for stroke. The competitive analysis indicated that compared with no anticoagulation, low-dose rivaroxaban (10 mg) remained significantly associated with a reduced risk of stroke (HR, 0.20; 95% CI, 0.05 to 0.78; P = 0.0201 for superiority); conventional anticoagulation also remained significantly associated with a reduced risk of stroke compared to no anticoagulation (HR, 0.21; 95% CI, 0.09 to 0.49; P = 0.0004 for superiority) (Table 2; Figure 2B).

There were 30 deaths from any cause in the non-anticoagulation group (16.96% per patient-year), 9 in the low-dose rivaroxaban group (5.36% per patient-year; HR, 0.28; 95% CI, 0.13 to 0.60; P = 0.0012 for superiority), and 40 in the conventional anticoagulation group (6.06% per patient-year; HR, 0.32; 95% CI, 0.19 to 0.52; P < 0.0001 for superiority) (Table 2; Figure 2D). The annualized rate of the death from cardiac causes was 11.86% in the non-anticoagulation group and 3.57% in the low-dose rivaroxaban group (HR, 0.28; 95% CI, 0.11 to 0.70; P = 0.0067 for superiority) and 3.64% in the conventional anticoagulation group (HR, 0.28; 95% CI, 0.15 to 0.52; P < 0.0001 for superiority). For cardiac death, non-cardiac death was treated as a competing risk. In competing analysis, low-dose rivaroxaban (10 mg) remained significantly associated with a reduced risk of cardiac death (HR, 0.31; 95% CI, 0.17 to 0.81; P = 0.0170 for superiority) and conventional anticoagulation also remained significantly associated with a reduced risk of cardiovascular death compared to no anticoagulation (HR, 0.28; 95% CI, 0.15 to 0.52; P < 0.0001 for superiority) (Table 2; Figure 2C).

Further direct comparison using the low-dose rivaroxaban group as the reference (lower section of the table) revealed no significant differences between the low-dose rivaroxaban and conventional anticoagulation groups in the risks of stroke (HR, 0.85, 95% CI, 0.23 to 3.11), all-cause death (HR, 1.14, 95% CI, 0.54 to 2.40), or cardiac death (HR, 1.02, 95% CI, 0.41 to 2.53). However, the risk of major bleeding was higher in the conventional anticoagulation group than in the low-dose rivaroxaban group, though the difference was not statistically significant (HR, 2.16, 95% CI, 0.99 to 4.73; P = 0.0546). In competing risk analysis, there was still no significant difference between the low-dose rivaroxaban and conventional anticoagulation groups in the risks of stroke (HR, 0.97, 95% CI, 0.25 to 3.85) and cardiac death (HR, 0.89, 95% CI, 0.34 to 2.32) (Table 3). However, in the multivariable Cox proportional hazards regression model and the competing risk model, the bleeding risk showed a decreasing trend in the low-dose rivaroxaban group, but the difference was not statistically significant (multivariable Cox proportional hazards regression model: HR, 2.16, 95% CI, 0.99 to 4.73; P = 0.0546; competing risk model: HR, 2.01, 95% CI, 0.93 to 4.12; P = 0.0856) (Table 3).

**TABLE 3:** Primary and secondary efficacy and safety end points in anticoagulation subgroups.

Multivariate cox regression analysis			
Outcome	Low-dose rivaroxaban	Conventional anticoagulation	
		Hazard ratio (95% Cl)	P value
Major bleeding	References	2.16 (0.99, 4.73)	0.0546
Stroke	References	0.85 (0.23, 3.11)	0.8014
All cause death	References	1.14 (0.54, 2.40)	0.7303
Cardiac death	References	1.02 (0.41, 2.53)	0.9679
Competing risk analysis			
Major bleeding	References	2.01 (0.93, 4.12)	0.0856
Stroke	References	0.97 (0.25, 3.85)	0.9687
Cardiac death	References	0.89 (0.34, 2.32)	0.8071

All adjusted for age, gender, hemoglobin, coronary heart disease, diabetes, hypertension, eGFR, ACEI. eGFR, estimated glomerular filtration rate; NSAID, nonsteroidal antiinflammatory drugs.

## 4 Discussion

This real-world study evaluated the effectiveness and safety of low-dose rivaroxaban (10 mg) in very elderly (≥80 years) patients with NVAF. The main findings are as follows: 1) Compared with no anticoagulation, low-dose rivaroxaban was associated with significantly lower risks of stroke, all-cause mortality, and cardiac death, with a magnitude of benefit comparable to conventional anticoagulation. 2) The risk of major bleeding in the low-dose rivaroxaban group and the conventional anticoagulation group were both higher than that in the no anticoagulation group. 3) In direct comparison, low-dose rivaroxaban was non-inferior to conventional anticoagulation in preventing stroke and death but was associated with a trend towards a lower risk of major bleeding, although this difference was not statistically significant.

Real-world registry data have shown that low-dose DOACs were prescribed in a high proportion of patients with AF (up to 30%–40%) compared with those patients recruited into the respective Phase III clinical trials. (Camm et al., 2019; Beyer-Westendorf et al., 2019). This inappropriate underdosing is mainly due to concerns about potential bleeding complications, especially in elderly patients (Yao et al., 2022). Even though DOAC underdosing is considered to be associated with a higher risk of thromboembolic stroke (Camm et al., 2020). Furthermore, elderly patients are underrepresented in major randomized controlled trials (RCTs) (Camm et al., 2019; Beyer-Westendorf et al., 2019). In the context of higher bleeding risks, the selection of dosages for the elderly remains a clinical challenge. Our findings provide evidence for this specific population. We found that in patients aged ≥80 years, rivaroxaban 10 mg was associated with effective prevention of thromboembolic events while showing a favorable trend toward reducing bleeding risk, thereby helping to alleviate clinicians’ concerns regarding bleeding in elderly patients and potentially improving the initiation and maintenance rates of anticoagulant therapy.

This study demonstrates that low-dose rivaroxaban (10 mg) provides a favorable net clinical benefit in very elderly patients, a finding consistent with existing evidence supporting dose adjustment in this population. An RCT compared reduced-dose edoxaban against a placebo in patients with AF aged ≥80 years and deemed ineligible for standard anticoagulation because of high bleeding risks. Low-dose edoxaban significantly reduced ischemic stroke or systemic embolism, albeit with a nonsignificant increase in major bleeding (Connolly et al., 2009). The meta-analysis conducted by Lin DS et al. suggest superior efficacies for rivaroxaban in preventing ischemic stroke or systemic embolism in patients aged ≥75 years compared with edoxaban and VKA (Lin et al., 2023). A real-world study focusing on Japanese patients with NVAF specifically indicated that dose-adjusted rivaroxaban (15 mg or 10 mg) resulted in stroke and major bleeding rates comparable to the VKA (Hori et al., 2012). Our results reinforce this perspective, confirming that in patients aged ≥80 years, low-dose (10 mg) rivaroxaban effectively prevents stroke and death while demonstrating a trend towards better safety, offering robust real-world evidence for implementing an individualized, low-dose anticoagulation strategy in this vulnerable population.

Bleeding risk remains a major deterrent to anticoagulation in the elderly. It was reported that the same dose of rivaroxaban resulted in higher bleeding rates in older patients compared to the young patients, suggesting that a dose adjustment may be preferred for older patients (Ren et al., 2024). Furthermore, a pharmacological review of rivaroxaban dosing emphasizes the recommendation for using 10 mg doses in elderly Chinese patients, providing a pharmacological rationale for the low-dose regimen (Zhang et al., 2023). In the direct comparison between low-dose rivaroxaban and conventional anticoagulation, we observed a numerical trend toward lower major bleeding risk with the low-dose regimen. However, this finding must be interpreted in the context of the substantial imbalance in group sizes (168 vs. 660), which resulted in limited statistical power to detect a significant difference between the two active treatment groups. The observed trend, while clinically intriguing, does not reach statistical significance and requires confirmation in larger, adequately powered studies. Despite this, this trend represents a clinically important finding. This suggests that dose reduction is a viable strategy to mitigate bleeding risk without compromising efficacy in selected high-risk elderly populations. While conventional anticoagulation has proven efficacy in stroke prevention, the bleeding cost is often higher in real-world elderly populations with multiple comorbidities. Our data indicate that low-dose rivaroxaban offers a favorable trade-off, showing a trend towards better safety.

### 4.1 Limitations

Our study has several limitations. First, it is a single-center observational study. Although we performed multivariate adjustments, unmeasured confounding factors cannot be completely ruled out. Second, the baseline characteristics were imbalanced, reflecting real-world clinical decision-making influenced by patients’ overall condition; we statistically adjusted for these imbalances to mitigate their impact. Third, the follow-up period was 12 months; the long-term efficacy and safety beyond 1 year warrant further investigation. Fourth, the notable imbalance in group sizes between the low-dose rivaroxaban (n =168 ) and conventional anticoagulation (n $\left.=660\right)$ groups substantially reduced the statistical power for direct comparisons, particularly for the safety outcome of major bleeding. The non-significant P-value for this comparison should be interpreted with caution, and the observed numerical trend requires validation in larger prospective cohorts or randomized controlled trials. Fifth, the follow-up period was limited to 12 months. While this duration is sufficient to capture early differences in safety outcomes such as major bleeding, it may be inadequate for fully assessing long-term outcomes such as stroke and mortality, which often accrue over years in patients with atrial fibrillation. Despite these limitations, a key strength of our study is its focus on a real-world, very elderly population (≥80 years) that is often underrepresented in clinical trials, thus providing valuable contemporary insights into the management of this growing patient group.

## 5 Conclusion

In patients aged ≥80 years with non-valvular atrial fibrillation, low-dose rivaroxaban (10 mg) was associated with significantly lower risks of stroke and death compared to no anticoagulation, with efficacy comparable to conventional anticoagulation. Furthermore, it demonstrated a promising safety profile, with a numerically lower risk of major bleeding than conventional anticoagulation, although this difference was not statistically significant in our cohort. This study provides robust real-world evidence suggesting that low-dose rivaroxaban may be a valuable option as an individualized antithrombotic strategy in this vulnerable, very elderly, high-risk population.

## Data Availability

The raw data supporting the conclusions of this article will be made available by the authors, without undue reservation.
